# Focal Pigmented Squamous Cell Carcinoma (pSCC) In Situ of the Nail: A Rare Case Emphasizing Diagnostic Vigilance

**DOI:** 10.7759/cureus.100087

**Published:** 2025-12-25

**Authors:** Sana Altaf, Haowei Han, Valerie Foy, Jacqueline Nikakis, Jay Dennett

**Affiliations:** 1 Dermatology, Touro College of Osteopathic Medicine, New York, USA; 2 Dermatology, St. John's Episcopal Hospital, New York, USA; 3 Dermatology, St. John’s Episcopal Hospital, New York, USA; 4 Dermatology, Larkin Community Hospital, Miami, USA; 5 Dermatology, NYU Langone Health, New York, USA

**Keywords:** malignant nail tumor, mohs surgery, nail, nail unit, squamous cell carcinoma, subungual

## Abstract

Pigmented squamous cell carcinoma (pSCC) of the nail unit is an uncommon condition that can mimic benign entities. Clinically, pSCC can present as longitudinal melanonychia, onycholysis, or verrucous plaques, potentially leading to diagnostic delays. The pigmented variant is particularly challenging to diagnose, as it may be mistaken for benign melanocytic lesions. Histologically, pSCC demonstrates atypical keratinocyte proliferation with pigmentation, and immunohistochemical analysis is crucial to exclude melanocytic involvement. This case report describes a 25-year-old immunocompetent female with Fitzpatrick type IV skin who presented with progressive nail discoloration and sensitivity of the right thumb, persisting for approximately one year. Despite the absence of a personal or family history of skin malignancy, clinical evaluation and subsequent biopsy revealed focal pSCC in situ with wart-like histopathological features, including full-thickness epithelial atypia, papillomatosis, hypergranulosis, and keratohyalin granule formation. The patient was referred to Moh’s micrographic surgery. This case underscores the importance of maintaining a broad differential diagnosis when evaluating persistent or evolving nail pigmentation, especially in patients without classic risk factors. The wart-associated histological changes suggest a possible link to human papillomavirus (HPV) infection, warranting further investigation into viral oncogenesis in nail unit SCC. Clinicians should maintain a high index of suspicion for atypical presentations, particularly in cases with progressive changes and associated pain, to ensure timely diagnosis and optimal management.

## Introduction

Pigmented squamous cell carcinoma (pSCC) of the nail unit is a rare but important pathology to consider in patients presenting with longitudinal melanonychia. While longitudinal melanonychia is more commonly associated with benign conditions such as melanocytic activation, nevi, and trauma, malignancies-including acral melanoma and pSCC-must be considered, particularly in cases with progressive changes, associated symptoms, or atypical features [1]. pSCC is characterized by hyperpigmented keratinocyte malignancy rather than melanocytic proliferation. The pathogenesis is not fully understood, though chronic ultraviolet exposure, human papillomavirus (HPV) infection, and prior trauma have been implicated. The presence of wart-associated histopathological features in some cases suggests a potential role of HPV, though direct causality remains to be established [2].

Previous reports of nail unit pSCC have predominantly described cases in older, immunosuppressed, or darker-skinned individuals [3]. The condition is often insidious in onset, leading to diagnostic delays, particularly when mistaken for benign pigmentary changes. In most reported cases, surgical excision remains the treatment of choice, with Mohs micrographic surgery utilized to ensure complete removal while preserving function [4]. Here, we present a case of pigmented SCC of the nail in a young, immunocompetent female with Fitzpatrick type IV skin. This case highlights the importance of maintaining a broad differential diagnosis for nail pigmentation and considering biopsy in cases with atypical or evolving features.

## Case presentation

A 25-year-old woman with a past medical history of thyroid cancer, post thyroidectomy, not on any medications, presented to our clinic with complaints of nail discoloration she had noted for about a year. She also stated that her nail has been increasingly sensitive to heat and pain and that more recently it has become red. The patient also acknowledged nail-biting but denied a prior history of a wart on the finger. In addition, she reported attempting to peel back her nail, removing some of the dark pigmentation, but noted that the discoloration reappeared. 

**Figure 1:**
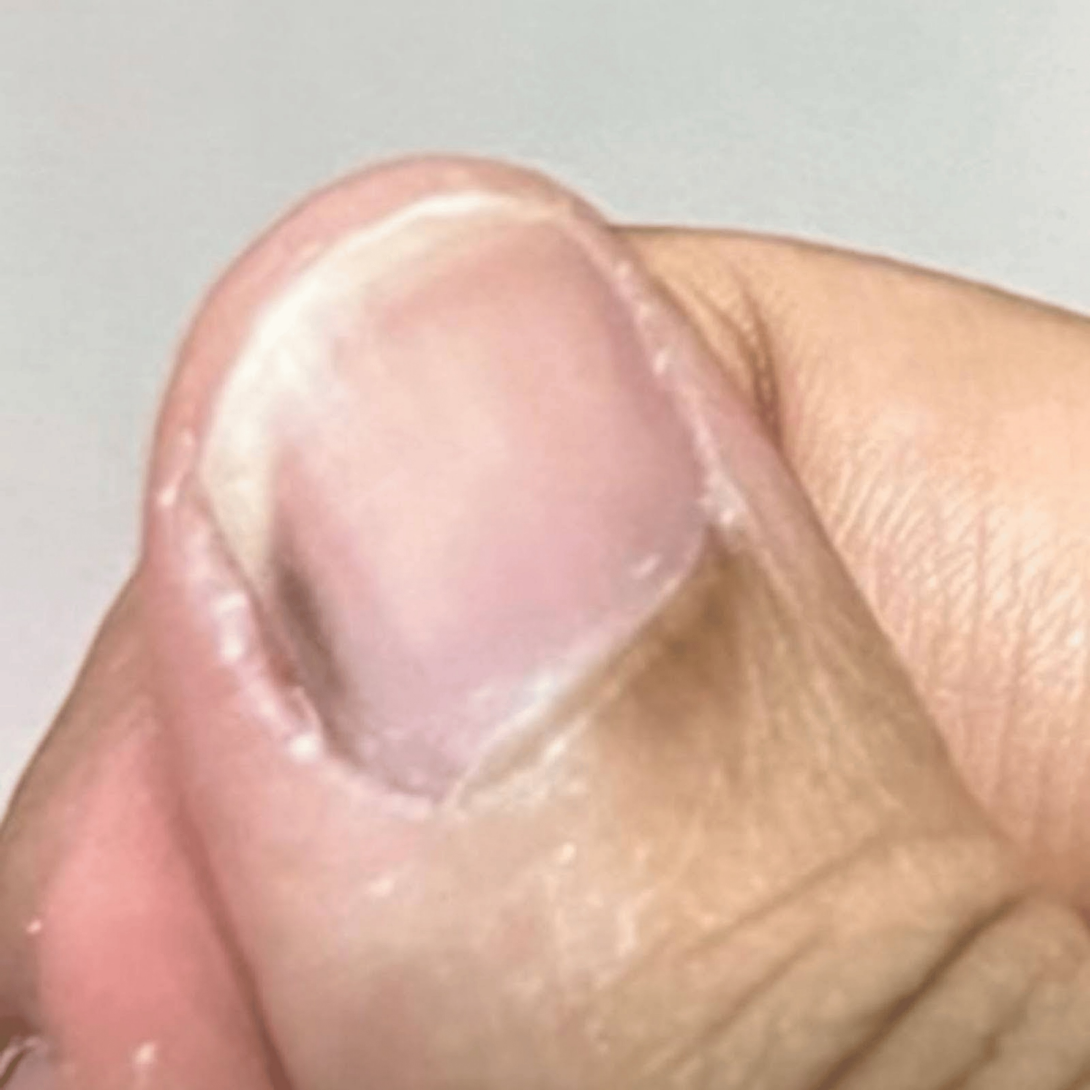
Linear dark brown approximately 1 mm-wide melanonychia was noted on the lateral right thumb without Hutchinson sign.

Family history was remarkable for anal cancer in her mother. No personal or familial history of either non-melanoma or melanoma skin cancers was reported. On examination, a linear dark brown approximately 1 mm-wide melanonychia was noted on the lateral right thumb with no Hutchinson sign present (Figure 1). A nail clipping and possible biopsy were planned in one month from presentation. A one-month period between presentation and follow-up was allowed for nail growth, and the patient was counseled to avoid painting, clipping, filing, and biting nails during this time.

**Figure 2:**
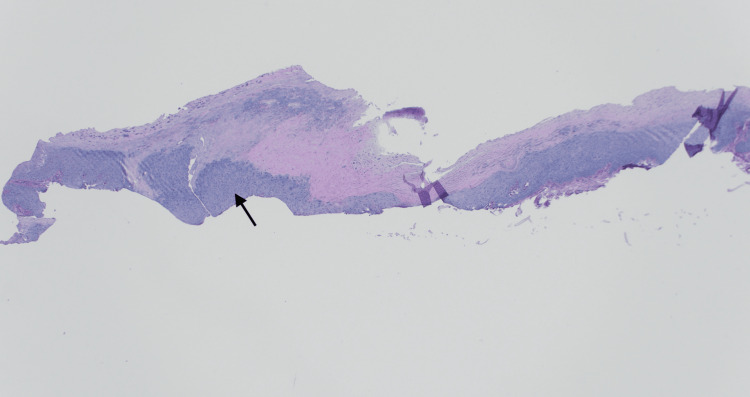
H&E x4. Full-thickness atypia with hyperkeratosis (arrow).

**Figure 3:**
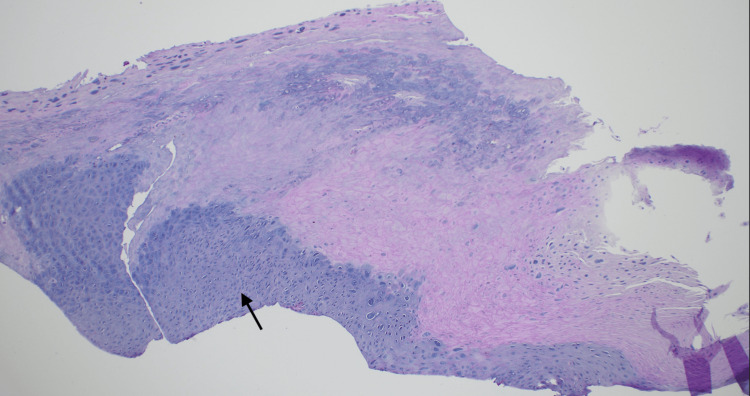
H&E x10. Full-thickness atypia with hyperkeratosis (arrow).

At follow-up, a shave biopsy was performed. Dermatopathology revealed focal pSCC in situ associated with a wart. Histopathologic examination demonstrated full-thickness atypia of the epithelium with features of SCC in situ (Figures 2, 3). In addition, wart-associated changes, including papillomatosis, hypergranulosis, and keratohyalin granule formation, were identified. Melan-A and SOX-10 immunohistochemical staining confirmed the absence of a melanocytic lesion. Fontana-Masson staining highlighted pigment deposition within the epithelium, nail plate, and associated hyperkeratosis.

**Figure 4:**
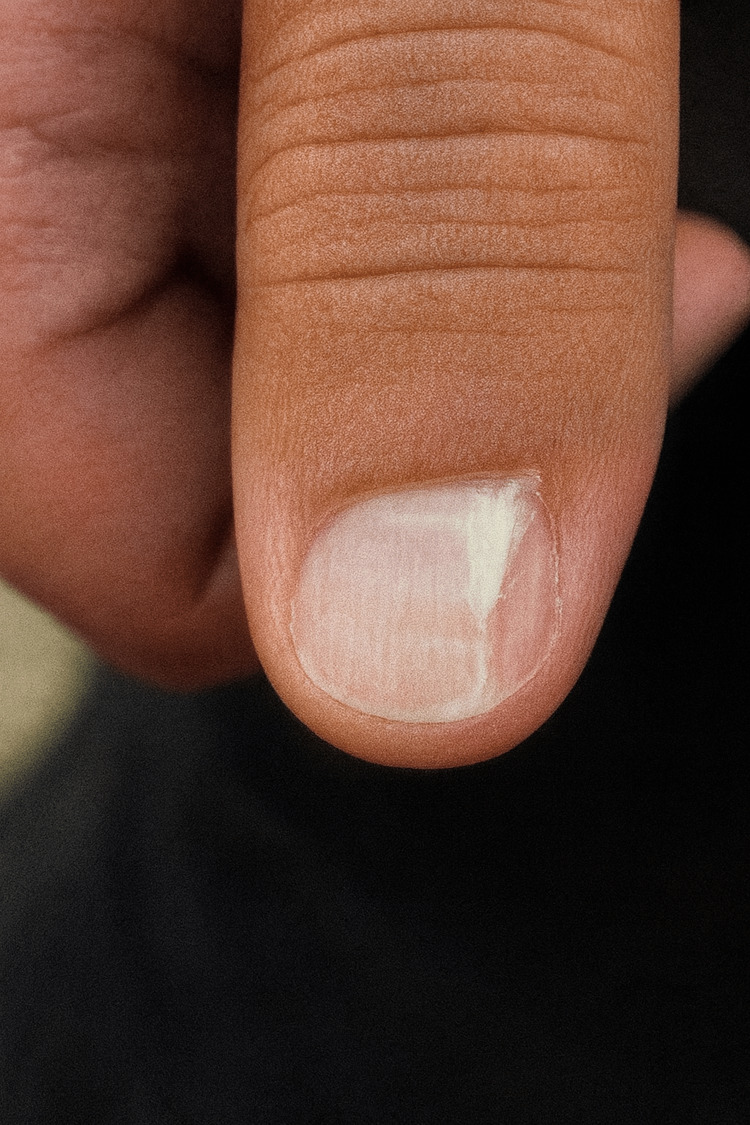
One-month post Mohs surgery.

The patient underwent Mohs micrographic surgery for complete excision of the lesion. She recovered well postoperatively, with no evidence of recurrence at the one-month follow-up (Figure 4). 

## Discussion

pSCC of the nail unit is a rare malignant tumor, often misdiagnosed as a benign lesion. However, it is the most common primary malignant neoplasm of the nail matrix, the tissue from which the nail grows. Symptoms can vary widely and include paronychia, dyschromia, onycholysis, and pain. The tumor may also mimic other conditions, such as melanoma, onychomycosis, subungual hemorrhage, or a viral wart. If there is a significant delay in diagnosis, invasiveness may occur, resulting in the need for digital amputation. The treatment for subungual SCC is typically determined by the tumor's extent. Lesion location within the high-risk H-zone, including sites such as the nail unit, can often be microscopically excised using Mohs micrographic surgery, which allows for minimal tissue removal and clean margins [4]. Many cases of nail pigmentation stem from benign causes such as trauma or fungal infection [5]. In this case, however, the persistent discoloration, sensitivity, and reappearance of pigmentation warranted further investigation. Histopathological analysis confirmed focal pSCC in situ with wart-associated changes, and immunohistochemistry excluded other melanocytic lesions [6]. 

SCC typically affects men between the ages of 50 and 59. It has been linked to various etiologic factors, including chronic infections, chemical or physical microtrauma, genetic disorders such as congenital ectodermal dysplasia, exposure to radiation, tar, arsenic, or minerals, prolonged sun exposure, immunosuppression, and a prior history of HPV infection [3]. Several studies suggest that mucosal HPV strains may contribute to malignancies in the nail unit. HPV DNA has been detected in 60-90% of subungual SCC cases, with over 60% of HPV-positive specimens associated with HPV 16 [7]. In our case, potential causative factors were ruled out through clinical history and physical examination.

Subungual melanoma often presents with overlapping features, such as longitudinal melanonychia. A few features of melanonychia that warrant further investigation include solitary or wider (>2 to 3mm) bands of longitudinal pigment or areas of bands that entirely lack pigment. Pigment on the periungual skin may also be a sign of concern [8]. In this case, the absence of Hutchinson's sign and negative immunohistochemical staining for melanocytic markers, including Melan-A and SOX-10, helped exclude melanoma as a diagnosis, while the triangular sign and solitary lesions on nails were suggestive of malignancy [3]. The wart-associated changes in this case suggest a possible link to HPV infection, which is often implicated in such lesions [7]. The patient’s nail-biting habit likely contributed to chronic trauma or provided a pathway for viral inoculation, potentially playing a role in malignant transformation. However, the exact contribution of these factors remains uncertain. The family history of anal cancer in the patient’s mother raises the possibility of an inherited susceptibility to squamous cell neoplasia.

When HPV testing is pursued, PCR is the most direct method for detecting viral DNA, and p16 staining can sometimes provide supportive evidence. Clinically, any single-digit nail change that persists, worsens over time, or becomes painful should lower the threshold for nail unit biopsy. Sampling matters: limited biopsies can miss deeper invasive disease, and SCC in situ has been reported to be upstaged to invasive SCC in 7.7-9.7% of cases, so the biopsy should be planned to capture the most suspicious area and adequate depth whenever possible [9]. Prognosis is usually good, with metastasis uncommon (about 2-3%), but recurrence is not always early; recurrences have been reported at two and even 10 years after Mohs surgery [10]. For that reason, long-term surveillance remains appropriate even after clear margins.

## Conclusions

The patient’s treatment with Mohs micrographic surgery achieved complete lesion removal, with no evidence of recurrence at follow-up. This case highlights a unique presentation of focal pSCC in situ of the nail unit, emphasizing the importance of evaluating persistent nail changes, especially in patients with a history of malignancy or concerning symptoms. It also emphasizes the importance of precise surgical intervention alongside patient education on nail care and avoiding further trauma to prevent recurrence. In addition, this case serves as a reminder for clinicians to maintain a high index of suspicion for atypical presentations in high-risk patients, ensuring timely diagnosis and management to optimize clinical outcomes and preserve nail function and aesthetics.
